# Transcriptomic profiling of two Pak Choi varieties with contrasting anthocyanin contents provides an insight into structural and regulatory genes in anthocyanin biosynthetic pathway

**DOI:** 10.1186/s12864-017-3677-7

**Published:** 2017-04-11

**Authors:** Lu Zhang, Bin Xu, Tao Wu, Yanfang Yang, Lianxue Fan, Muxuan Wen, Jiaxin Sui

**Affiliations:** 1 College of Horticulture, Northeast Agricultural University, 59 Mucai street, 150030 Harbin, People’s Republic of China; 2 College of Agro-grassland Science, Nanjing Agricultural University, 1 Weigang, 210095 Nanjing, People’s Republic of China; 3 State Key Laboratory of Tree Genetics and Breeding, The Research Institute of Forestry, Chinese Academy of Forestry Sciences, 100091 Beijing, People’s Republic of China

**Keywords:** Anthocyanin, *Brassica campestris* L. *ssp. chinensis* L. Makino, Transcriptome, RNA-Seq

## Abstract

**Background:**

The accumulation of anthocyanin in horticultural crops not only improves their stress tolerances but also their nutritional values. Many key regulatory and structural genes in anthocyanin biosynthesis have been identified in model plants, but limited information is available for non-model plant species featured with colored leaves. In this study, two Pak Choi varieties with green or purple leaves were selected to analyze the anthocyanin biosynthesis through RNA-Seq.

**Results:**

A total of 2475 unigenes were differentially expressed between these tested varieties, including 1303 down-regulated and 1172 up-regulated genes in the purple-leafed one. The reliability of the RNA-Seq was further confirmed by using real-time quantitative PCR. Kyoto Encyclopedia of Genes and Genomes enrichment analysis of the differentially expressed genes revealed ‘flavonoid biosynthesis’ was the only enriched pathway in the purple-leafed variety: In the pathway of phenylpropanoid metabolism, *Bra017210*, *Bra039777*, and *Bra021637* were expressed at higher levels in the purple-leafed variety;　among the early anthocyanin biosynthetic genes, *Bra037747* transcripts were only detected in the purple-leafed variety but not in the green-leafed one; among the late anthocyanin biosynthetic genes, *Bra027457*, *Bra013652*, *Bra019350*, *Bra003021*, *Bra035004*, and *Bra038445* were all up-regulated in purple-leafed variety; and genes encoding anthocyanin-related transcription factors, such as *Bra016164*, and genes encoding anthocyanin transportation, such as *GST F12*, were also identified as up-regulated ones in the purple-leafed variety.

**Conclusions:**

The current result provided a valuable insight into the anthocyanin accumulation in the purple-leafed variety of Pak Choi and a bioinformatic resource for further functional identification of key allelic genes determining the difference of anthocyanin content between Pak Choi varieties.

**Electronic supplementary material:**

The online version of this article (doi:10.1186/s12864-017-3677-7) contains supplementary material, which is available to authorized users.

## Background

Most autotrophic higher plants in temperate regions are featured with leaf coloration during the progression of leaf senescence in autumn or under stress conditions mainly due to the accumulation of anthocyanins [1]. However, there are plants having inherent colored leaves (e.g. red or purple) during their entire life cycles. Horticultural crops with high anthocyanin contents are considered valuable for the health-promoting effect of anthocyanins [2]. The accumulation of anthocyanin is also proposed as an important strategy for plant tolerance against abiotic or biotic stresses at least partially through its photoprotective function against excessive sunlight and its antioxidant function for mitigating the signal transduction of and the damage effect of reactive oxygen species [3].

The anthocyanin biosynthesis pathway has been intensively studied in model plant Arabidopsis [4], which is initiated from branches of the flavonoid pathway: starting from the conversion of phenylalanine to cinnamic acid by phenylalanine ammonia-lyase (PAL), and then sequentially catalyzed by cinnamate 4-hydroxylase (C4H), 4-coumaroyl:CoA-ligase (4CL), chalcone synthase (CHS), chalcone isomerase (CHI) to form naringenin. Naringenin can be further catalyzed by flavanone 3-hydroxylase (F3H), dihydroflavonol 4-reductase (DFR), and anthocyanidin synthase (ANS/LDOX) to form pelargonidin [4–6], or catalyzed by two other enzymes flavonoid 3′-hydroxylase (F3′H) or flavonoid 3′5′-hydroxylase (F3′5′H) to hydroxylate the B-ring of dihydrokaempferol (DHK) into dihydroquercetin or dihydromyricetin that eventually forms cyanidin or delphinidin, respectively. In another branch, naringenin can be directly catalyzed by DFR and ANS to form apigenidin. Finally, these unstable anthocyanidins are glycosylated immediately to form anthocyanins [7]. Despite of the fact that this biosynthetic pathway is relatively conserved and finely characterized in model plants, deep insights are just recently gained into its upstream regulatory mechanism.

The anthocyanin biosynthesis pathway genes were regulated by transcription factors, such as the conserved MBW complex composed of MYB, basic Helix-Loop-Helix (bHLH) and WD40 subunit proteins in higher plants [8–10]. This MBW complex played essential roles in the synergistic regulation of anthocyanin accumulation through inhibiting or activating the expression of related genes [11–14], and this complex *per se* is repressed by MYBL2 and JAZ family proteins [15]. Furthermore, this MYBL2-JAZ repressor complex is sequestered by DELLA proteins [15]. This JAZ-DELLA-MYBL2 module upstream of the MBW complex together mediated abiotic stress-caused anthocyanin accumulation in *Arabidopsis* [15]. While the transferability of this knowledge into non-model plants needs to be further tested, it is of particular interest to understand the regulatory mechanism of persistent accumulation of anthocyanin at high levels in horticultural plants where high contents of anthocyanins are often desirable.

Pak Choi (*Brassica Campestris* L. *ssp. chinensis* L. Makino), one of the most consumed vegetables in China and phylogenetically close to Arabidopsis contains different varieties with highly varied levels of anthocyanin [16, 17], which makes Pak Choi an ideal plant system for studying the mechanism underlying persistence accumulation of anthocyanin. The objective of this study was to identify and characterize key genes and regulating elements involved in leaf anthocyanin accumulation in Pak Choi through RNA-Seq with *Brassica rapa* L. spp. *pekinensis* as reference.

## Methods

### Plant materials

Two varieties of Pak Choi with different leaf colors were selected in this study: ‘Jingguan’ with green leaves (abbreviated as ‘G’ in this study), and ‘Zizuan’ with purple leaves (abbreviated as ‘P’). Seeds of ‘G’ were from Beijing Vegetable Research Center, Beijing Academy of Agriculture and Forestry Sciences, and those of ‘P’ were from Beijing Dongsheng Seed Company. The seeds were sown in pots at 25 °C in July 2015 in a modern greenhouse located in Chinese Academy of Forestry Sciences, Beijing, China. When the seedlings had five leaves in total, the third leaf from the bottom was sampled and stored at -80 °C before further processing. Six biological replicates were applied for anthocyanin content measurements, and three replicates for the RNA-Seq analysis.

### Analysis of total anthocyanin

Fresh leaf tissue (0.2 g) was collected for the measurement of anthocyanin content. In brief, anthocyanins were extracted in 10 ml acidified methanol (99 CH_3_OH:1HCl, v/v) at 4 °C for 24 h in the dark, clarified by centrifugation at 12,000 × *g* for 2 min, and the absorbance of supernatants was determined using a UV-visible spectrophotometer (UV-2550, Shimadzu, Japan) from optical density (OD) at 530 and 600 nm. Anthocyanin concentrations were expressed as U∙g^-1^, where U was calculated as (OD530–OD600)/0.1 [18].

### RNA extraction and library preparation for transcriptome analysis

A total of 3 μg RNA *per* sample was applied for the RNA sample preparations. Sequencing libraries were generated using NEBNext® Ultra™RNA Library Prep Kit for Illumina® (NEB, USA) according to manufacturer’s instructions. By using poly-T oligo-attached magnetic beads, mRNA was purified from total RNA. Fragmentation was operated using divalent cations under elevated temperature in NEBNext First Strand Synthesis Reaction Buffer (5X). First strand cDNA was synthesized using random hexamer primer and M-MuLV Reverse Transcriptase (RNase H^-^). Second strand cDNA synthesis was then performed using DNA Polymerase I and RNase H. Remaining overhangs were converted into blunt ends via exonuclease/polymerase activities. After adenylation of 3′ ends of DNA fragments, NEBNext Adaptor with hairpin loop structure were ligated to be ready for hybridization. To select cDNA fragments of 150 ~ 200 bp in length, the library fragments were purified with AMPure XP system (Beckman Coulter, Beverly, USA). Then 3 μl USER Enzyme (NEB, USA) was used with size-selected, adaptor-ligated cDNA at 37 °C for 15 min followed by 5 min at 95 °C before PCR. Then PCR was performed with Phusion High-Fidelity DNA polymerase, Universal PCR primers and Index (X) Primer. In the end, PCR products were purified (AMPure XP system) and the assessment of library quality was conducted on the Agilent Bioanalyzer 2100 system. The library preparations were sequenced on an Illumina Hiseq 2500 platform and 125 bp paired-end reads were generated.

### Analysis of Illumina sequencing results

Clean data (clean reads) were obtained by deleting reads containing poly-N, reads containing adapters, and low quality reads from raw data. At the same time, Q20, Q30, GC-content of the clean data were calculated. Based on clean data of high quality, the downstream analyses were conducted.

### Quantification of gene expression levels and differential expression analysis

Reference genome and gene model annotation files were downloaded from *Brassica* database website directly [http://brassicadb.org/brad/] [19]. Clean data were mapped back onto the reference genome using TopHat v2.0.12. The HTSeq v0.6.1 software was used to count the reads numbers mapped onto each gene. And the expected number of Fragments *per* Kilobase of transcript sequence *per* Millions base pairs sequenced (FPKM) of each gene was calculated based on the length of the gene and reads count mapped to this gene. Differential expression analysis of two varieties (three biological replicates *per* variety) was performed using DEseq program [20]. Genes with an adjusted *P*-value <0.05 found by DESeq were considered as differentially expressed. Gene Ontology (GO) enrichment analysis of the differentially expressed genes (DEGs) was conducted by the GO seq R packages based Wallenius non-central hyper-geometric distribution [21], which can adjust for gene length bias in DEGs. KOBAS 2.0 [22] was used to test the statistical enrichment of DEGs in Kyoto Encyclopedia of Genes and Genomes (KEGG) pathways.

### Real-time quantitative RT-PCR (RT-qPCR) assay

The expression patterns of nine genes involved in the anthocyanin pathway (Gene ID: *Bra*013652, *Bra*019350, *Bra*027457, *Bra*037747, *Bra*017520, *Bra*017523, *Bra*026967, *Bra*016164, and, *Bra*032635) were analyzed using qRT-PCR. cDNA was synthesized using ReverseTra Ace qPCR RT Kit (Toyobo, Japan) according to the manufacturer’s recommendation. The reverse transcription reaction system contained 2 μL 5 × RT buffer, 0.5 μL primer mix, 0.5 μL RT enzyme mix, 2 μL RNA template, 5 μL ddH_2_O. Three biological replicates were applied for each gene expression analysis. Gene-specific primers were designed according to the reference unigene sequence using the online tool [GenScript Real-time PCR (TaqMan) Primer Design, https://www.genscript.com/ssl-bin/app/primer]. The cDNA diluted to 100 ng/μL was used for qPCR assay with each gene-specific primers and SYBR® Green Real time PCR Master Mix (Toyobo, Japan) on the Bio-Rad iQ5 real time system. Reactions were performed at 96 °C for 1 min, 40 cycles of 95 °C for 15 s, 60 °C for 15 s and 72 °C for 45 s. All primers for RT-qPCR are listed (Additional file 1: Table S1).

## Results

### Quantification of anthocyanin contents of two Pak Choi varieties

Two Pak Choi varieties were used in this study with contrasting colors that the purple variety (hereafter abbreviated as ‘P’) had 11.703 U∙g^-1^ of anthocyanins while the green one (hereafter abbreviated as ‘G’) only had 0.320 U∙g^-1^ (Table 1).

**Table 1:** Anthocyanin contents of two Pak Choi varieties with different leaf colors

Variety	Anthocyanin contents (U g^-1^ Fresh weight)
Green-leafed variety ‘G’	0.320 ± 0.054 b
Purple-leafed variety ‘P’	11.703 ± 1.360 a

Values are Mean ± SD of 6 replications. Values followed by different letters indicate significant difference at *P* < 0.05

### RNA-sequencing of two Pak Choi varieties with different leaf colors

**Fig. 1:**
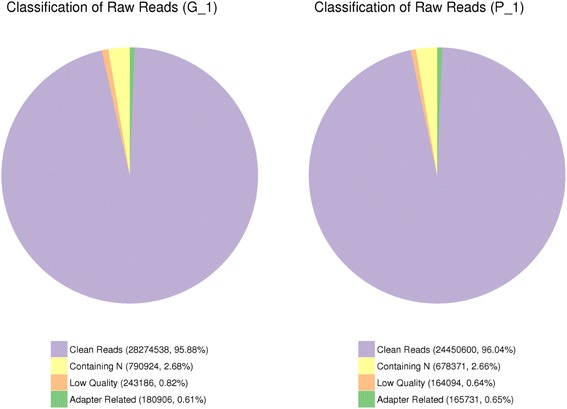
The quality of raw reads of *green-leafed* variety (‘G’) and *purple-leafed* variety (‘P’) of Pak Choi using RNA-Seq

Leaves at the same growth stage were sampled from these two varieties and their transcriptomes were profiled using Illumina sequencing. In total, 172,380,454 and 168,368,500 raw reads were generated from the ‘G’ and ‘P’ libraries, respectively (Table 2). To ensure the quality of the libraries, adaptor reads, ambiguous reads and low-quality reads were removed (Fig. 1). Finally, 165,289,336 and 161,562,282 clean reads were obtained for ‘G’ and ‘P’, respectively (Table 2). A total of 67.63% reads in green-leafed variety and 69.89% reads in purple-leafed variety were mapped in the *Brassica* database (BRAD). On average, 66.3% and 68.57% mapped reads were uniquely mapped to the database (Table 2).

**Table 2:** Summary of sequences analysis and RNA-Seq data

Sample name	Green-leafed variety ‘G’	Purple-leafed variety ‘P’
Raw reads	172,380,454	168,368,500
Clean reads	165,289,336	161,562,282
Clean bases	24.79G	24.24G
Error rate (%)	0.01	0.01
Q20 (%)	97.97	98.01
Q30 (%)	94.95	94.99
GC content (%)	48.03	47.91
Total mapped	111,825,953 (67.63%)	112,860,042 (69.89%)
Multiple mapped	2,211,063 (1.33%)	2,111,248 (1.33%)
Uniquely mapped	109,614,890 (66.30%)	110,748,794 (68.57%)
Reads map to ‘ + ’	54,945,243 (33.24%)	55,565,180 (34.40%)
Reads map to ‘-’	54,669,647 (33.07%)	55,183,614 (34.16%)
Non-splice reads	63,472,079 (38.40%)	65,056,741 (40.27%)
Splice reads	46,142,811 (27.90%)	45,692,053 (28,29%)

Q20: The percentage of bases with a Phred value >20

Q30: The percentage of bases with a Phred value >30

### Differentially expressed genes (DEGs) between the two Pak Choi varieties

**Fig. 2:**
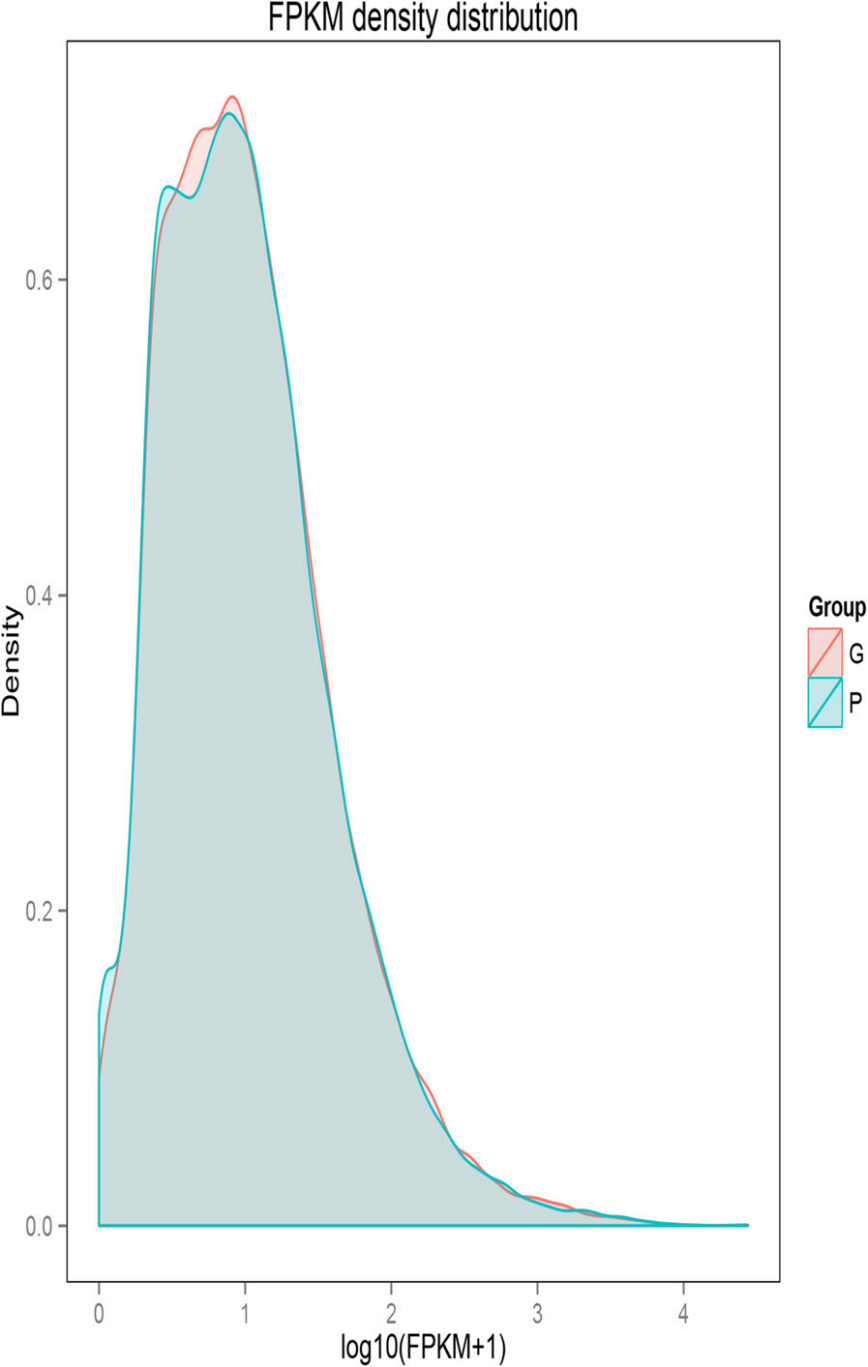
Frequency distribution of *green-leafed* variety (‘G’) and *purple-leafed* variety (‘P’) of Pak Choi by fragments per kilobase per million

Normalized-FPKM (fragments *per* kilobase *per* million) were used to quantify the transcript levels. The FPKM of the two varieties (‘G’ and ‘P’) were almost the same (Fig. 2) for unbiased analysis of their transcript profiles. The DEGs were analyzed to identify candidate genes related to the synthesis of anthocyanins. DEGs (padj < 0.05) were defined as genes that were significantly enriched or depleted in one variety relative to the other one. Between ‘G’ and ‘P’, a total of 2475 DEGs were identified, including 1303 down-regulated and 1172 up-regulated DEGs in ‘P’ (Additional file 2: Table S2).

### Verification of RNA-Seq data by real-time quantitative RT-PCR

To confirm the reliability of the RNA-Seq data, the relative expression levels of nine DEGs were examined by real-time quantitative PCR (RT-qPCR). Among the tested genes, one encoded a member of the oxidoreductase family protein, four transcription factor genes, and four anthocyanin biosynthetic enzymes. Consistent with the RNA-Seq data, relative expression levels of all selected genes were higher in the purple-leafed variety than in the green-leafed variety. This result further confirmed the reliability of the RNA-Seq data (Table 3).

**Table 3:** The expression patterns of selected genes using real-time quantitative RT-PCR and RNA-Seq

Gene	RT-PCR		RNA-Seq (FPKM)		Gene annotation
	‘G’	‘P’	‘G’	‘P’	
Bra013652	0.88 ± 0.16 b	179.31 ± 87.33 a	79.26	11936.77	Leucoanthocyanidin dioxygenase
Bra019350	1.00 ± 0.05 b	2.72 ± 0.41 a	23.62	4969.47	Leucoanthocyanidin dioxygenase
Bra027457	1.35 ± 0.47 b	23.61 ± 2.38 a	67.91	15507.10	Dihydroflavonol-4-reductase
Bra037747	1.13 ± 0.11 b	3.08 ± 0.47 a	0	26.11	Flavonol synthase/flavanone 3-hydroxylase
Bra017520	1.18 ± 0.31 b	14.76 ± 3.48 a	0.94	21.80	Basic helix-loop-helix (bHLH) family protein
Bra017523	1.11 ± 0.14 b	28.85 ± 0.80 a	16.31	540.00	Basic helix-loop-helix (bHLH) family protein
Bra026967	1.12 ± 0.14 b	8.88 ± 1.45 a	0	12.00	oxidoreductase family protein
Bra016164	0.93 ± 0.11 b	193.23 ± 8.24 a	5.87	865.19	MYB08 Myb-related protein 308
Bra032635	0.01 ± 0.00 b	1.04 ± 0.03 a	2.84	373.93	ETC1 (ENHANCER OF TRY AND CPC 1); DNA binding / transcription factor

The relative quantitation of gene expression was conducted via the 2 ^− ΔΔCt^ method, with actin as an endogenous reference. Data from three biological replicates were used to calculate the mean and standard deviation in DPS based on Student’s *t*-test. Values followed by different letters indicate significant difference at *P* < 0.05. ‘*G*’: Green-leafed variety; ‘*P*’: Purple-leafed variety

### Functional classification of DEGs

**Fig. 3:**
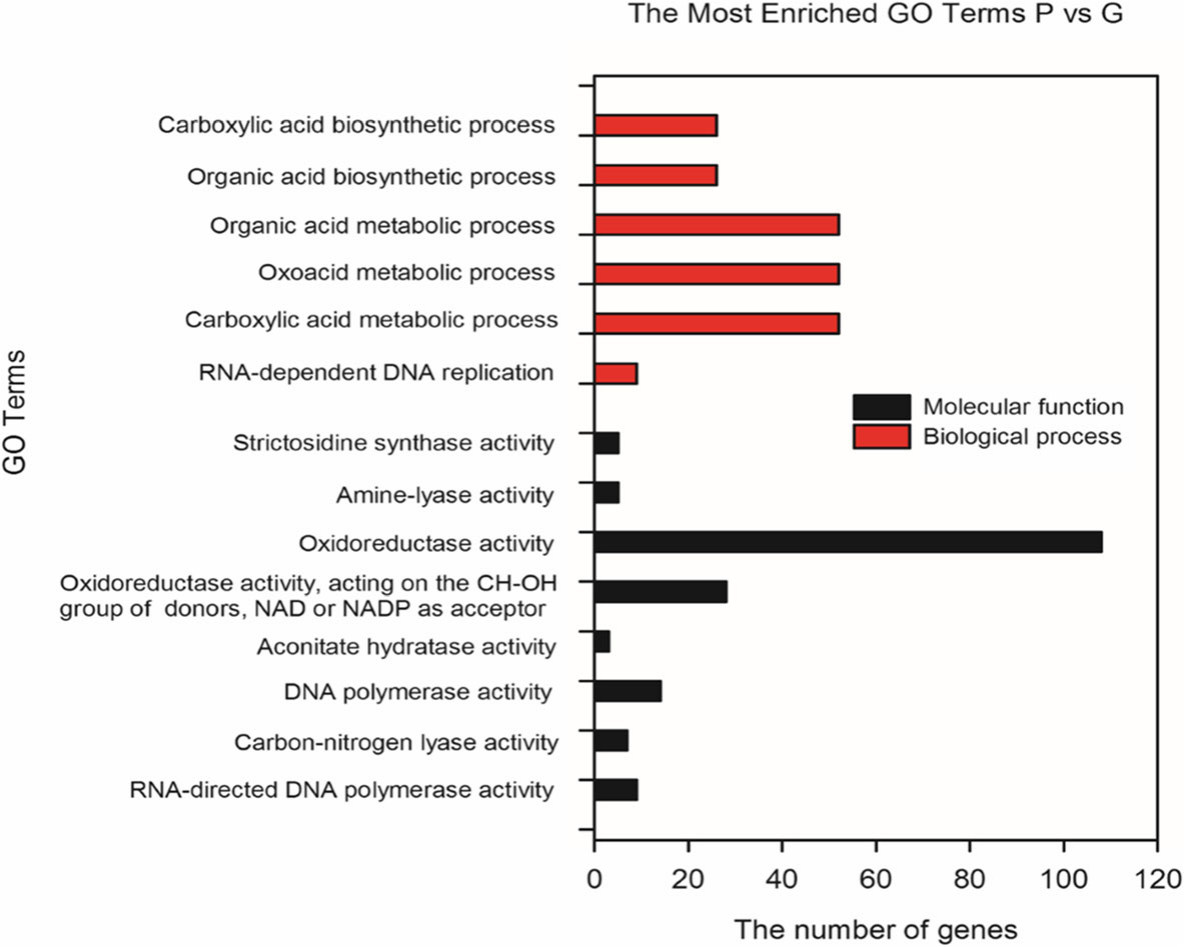
Go enrichment of up-regulated genes (corrected *p*-value < 0.5) in leaves of *purple-leafed* variety (‘P’) compared with those of *green-leafed* variety (‘G’) of Pak Choi

**Fig. 4:**
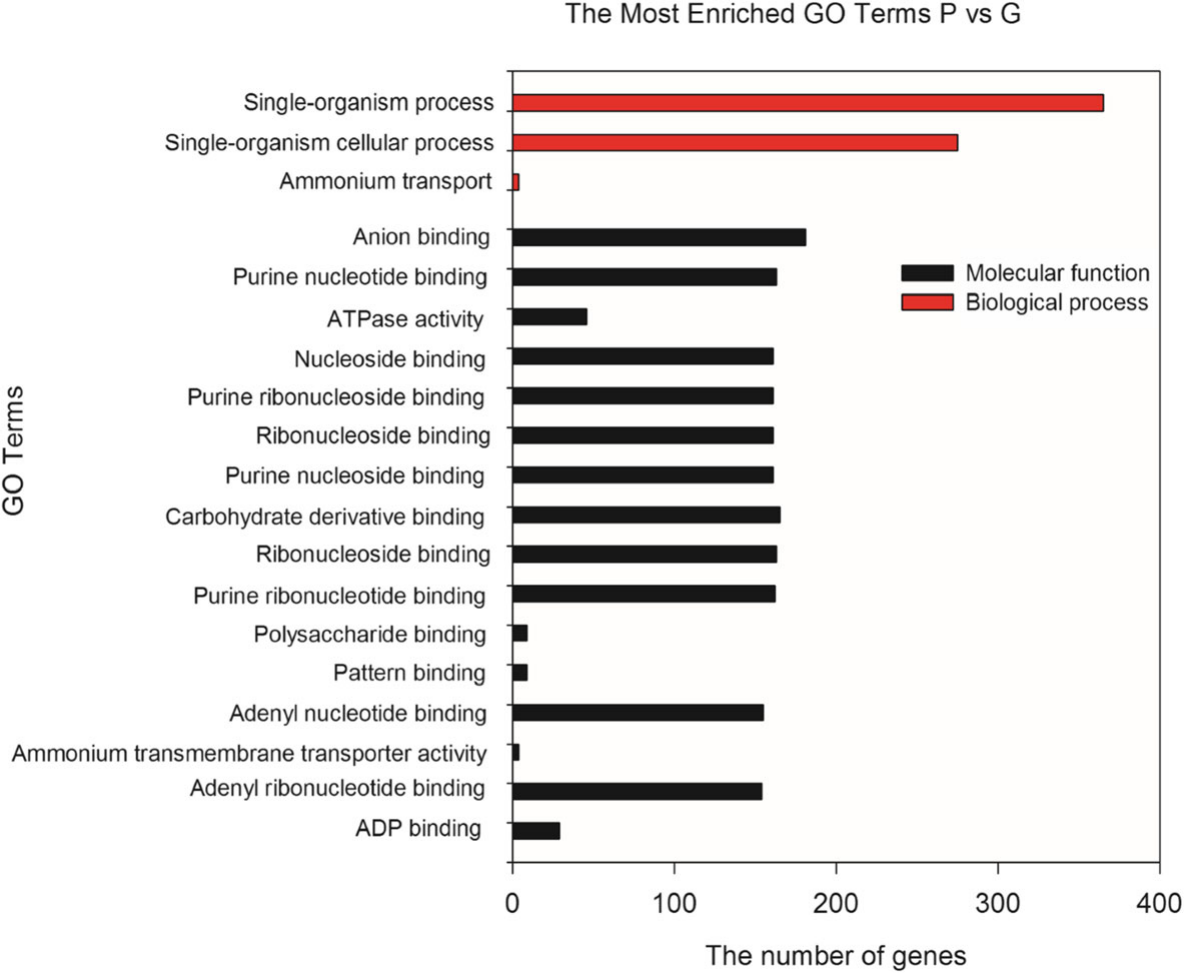
Go enrichment of down-regulated genes (corrected *p*-value < 0.2) in leaves *purple-leafed* variety (‘P’) compared with those of *green-leafed* variety (‘G’) of Pak Choi

GO classifications were conducted in order to predict DEGs’ functions. GO enrichment analysis (corrected *p*-value < 0.5) of the up-regulated DEGs in the ‘P’ suggested their variety-specific functions. In the category of biological processes, the highly enriched DEGs in ‘P’ included those involved in ‘carboxylic acid metabolic process’, ‘oxoacid metabolic process’, ‘organic acid metabolic process’, ‘organic acid biosynthetic process’, ‘carboxylic acid biosynthetic process’ and ‘RNA-dependent DNA replication’. In the molecular function category, the ‘oxidoreductase activity’ and ‘oxidoreductase activity, acting on the CH-OH group of donors, NAD or NADP as acceptor’ were the mostly highly enriched (Fig. 3). GO analysis (corrected *p*-value < 0.2) was also conducted for the down-regulated DEGs in ‘P’ (Fig. 4). In the category of biological processes, ‘single-organism process’ and ‘single-organism cellular process’ were the mostly highly enriched ones; and in the category of molecular function, the top enriched terms were ‘anion binding’, and ‘carbohydrate derivative binding’, (Fig. 4).

**Fig. 5:**
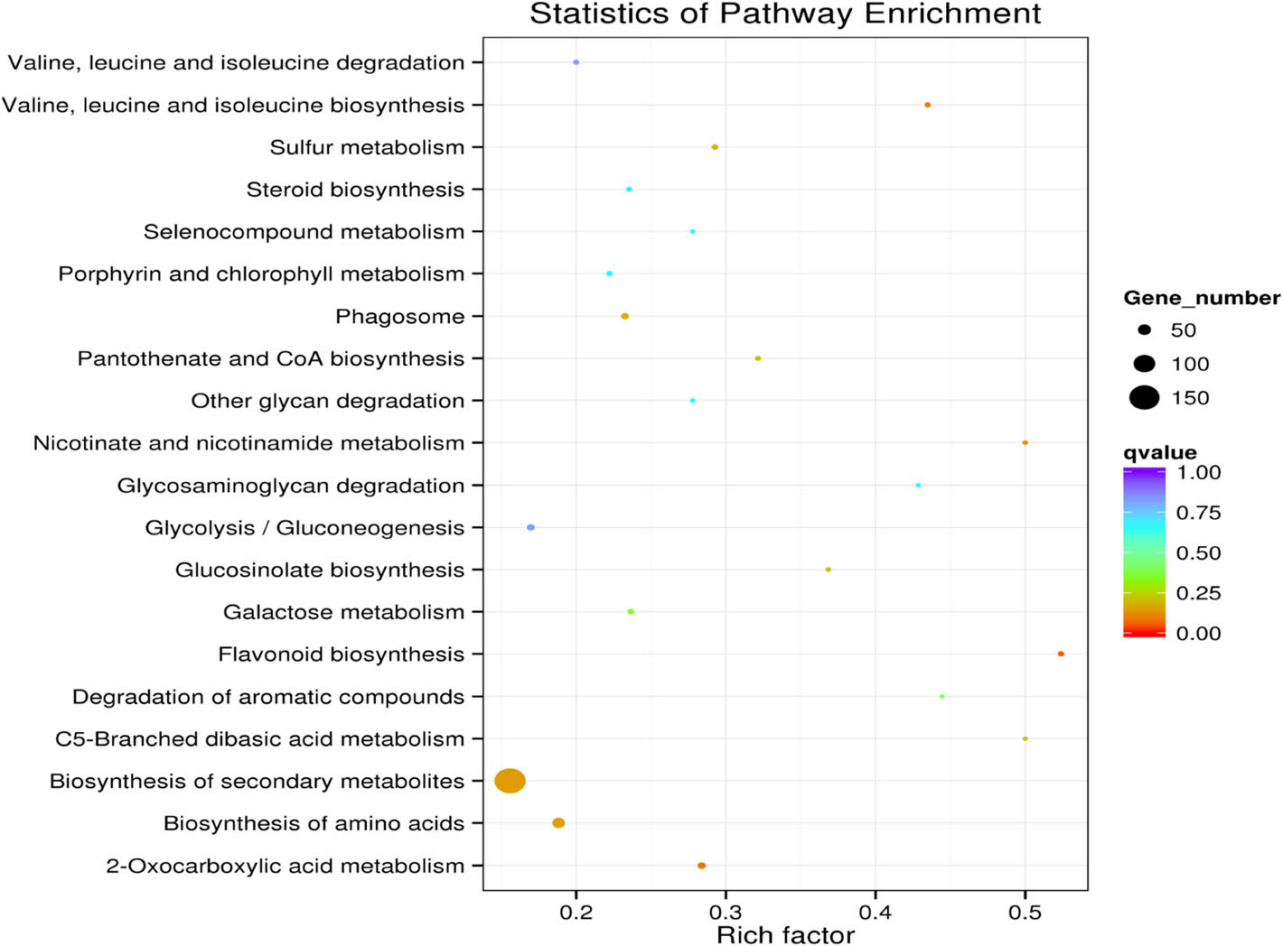
KEGG enrichment of differentially expressed genes in leaves between *green-leafed* variety (‘G’) and *purple-leafed* variety (‘P’) of Pak Choi

**Fig. 6:**
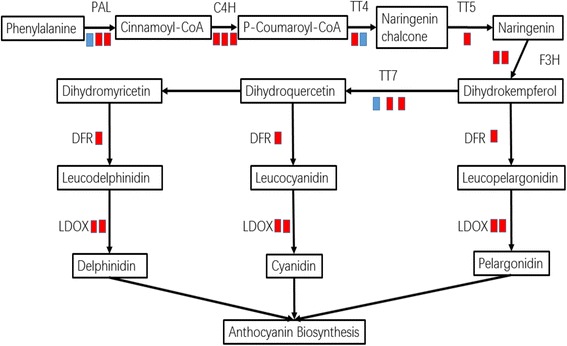
Differential expressed genes predicted to be involved in the flavonoid biosynthesis pathway. *Red block* means the up-regulated genes and *blue block* means the down-regulated genes in *purple-leafed* variety (‘P’) compared with *green-leafed* variety (‘G’) of Pak Choi

The result of KEGG pathway enrichment analysis for DEGs showed that the only enriched pathway of DEGs (corrected-*P* value is almost 0.05) was ‘flavonoid biosynthesis’ [KEGGmap00941 (http://www.genome.jp/dbget-bin/www_bget?map00941)] (Fig. 5), and there were 11 DEGs between ‘P’ and ‘G’ involved in this pathway (Fig. 6). Most of these genes were up-regulated in purple-leafed variety. For examples, the biosynthetic genes of anthocyanins, *Bra027457*, *Bra013652*, *Bra019350*, *Bra003021*, *Bra035004*, and *Bra038445* were all up-regulated in ‘P’ (Table 4). In the phenylpropanoid metabolism, *Bra017210*, *Bra039777*, and *Bra021637* were expressed at a higher level in ‘P’ than those in ‘G’. However, *Bra006985* had higher expression in ‘G’ than those in ‘P’. For biosynthetic genes in upstream of the anthocyanin biosynthesis pathway, *Bra037747* was expressed in ‘P’ rather than in ‘G’, yet *Bra029212* was expressed at a lower level in ‘P’ than those in ‘G’. Genes involved in anthocyanin transport were also identified, and the pair of allelic genes (*Bra008570 and Bra023602*) encoded *Glutathione S-transferase F12* were both significantly up-regulated in the purple-leafed variety (Table 4).

**Table 4:** The expression patterns of related genes for anthocyanin biosynthesis and transportation

			FPKM	
	Gene ID	Gene annotation	‘G’	‘P’
Biosynthetic genes in phenylpropanoid pathway	Bra017210	Phenylalanine ammonia-lyase 1	3566.60	8616.42
	Bra039777	Phenylalanine ammonia-lyase 2	930.36	7009.32
	Bra006985	Phenylalanine ammonia-lyase 2	3299.06	18.24
	Bra031266	4-coumarate--CoA ligase 2	216.25	624.71
	Bra021637	Transcinnamate 4-monooxygenase	939.76	4518.74
Early Biosynthetic Genes	Bra009101	Probable chalcone--flavonone isomerase 3	1281.89	5917.44
	Bra037747	Flavonol synthase/flavanone 3-hydroxylase	0	26.11
	Bra029212	Flavonol synthase 3	790.26	328.98
Late Biosynthetic Genes	Bra027457	Dihydroflavonol-4-reductase	67.91	15507.10
	Bra013652	Leucoanthocyanidin dioxygenase	79.26	11936.77
	Bra019350	Leucoanthocyanidin dioxygenase	23.6186	4969.47
	Bra035004	Anthocyanidin 3-O-glucosyltransferase	10.15	1736.71
	Bra003021	Anthocyanidin 3-O-glucosyltransferase	5.63	1011.59
	Bra038445	UDP-glycosyltransferase 75C1	56.36	10314.18
	Bra011292	Caffeic acid 3-O-methyltransferase 1	19.03	276.83
	Bra034600	Caffeoyl-CoA O-methyltransferase 1	1080.65	6484.01
	Bra003009	Quercetin 3-O-methyltransferase 1	8040.55	568.33
	Bra003707	Caffeic acid 3-O-methyltransferase OS	159.46	1.43
Transport genes in anthocyanin pathway	Bra009033	Protein TRANSPARENT TESTA 12	22.96	3.61
	Bra016610	ATPase 10, plasma membrane-type	0.30	63.40
	Bra024452	ATPase 1, plasma membrane-type	6.84	199.33
	Bra008570	Glutathione S-transferase F12	12.02	2134.23
	Bra023602	Glutathione S-transferase F12	195.28	2391.95

‘*G*’: Green-leafed variety; ‘*P*’: Purple-leafed variety

Transcription factors play essential roles in the transcriptional regulation of structural genes in anthocyanin biosynthesis. Therefore candidate transcription factors potentially involved in anthocyanin biosynthesis were further pinpointed in the RNA-Seq analysis. It was found that *Bra036145* was suppressed, while *Br*a037887, *Bra016164* and *Br*a039283 were all up-regulated in purple-leafed variety (Table 5). Yet, *Bra011772* was expressed at significantly lower level in purple-leafed variety than green-leafed one. The predicated functions of these DEGs according to their Arabidopsis orthologs were shown in Table 5.

**Table 5:** Selected genes about transcription factor with altered expression

			FPKM	
Group	Gene ID	Gene Annotation	‘G’	‘P’
Transcription factor	Bra016164	MYB08 Myb-related protein 308	5.87	865.19
	Bra036145	MYB12 Transcription factor MYB12	46.07	0
	Bra039283	Transcription factor CPC	0.98	42.77
	Bra037887	TT8 Transcription factor TT8	3.24	917.07
	Bra011772	LBD39	101.15	19.33

‘*G*’: Green-leafed variety; ‘*P*’: Purple-leafed variety

## Discussion

The increased accumulation of anthocyanin in leafy vegetables improves their commercial and healthy values. Yet, there are limited studies on mechanisms underlying the anthocyanin biosynthesis in leafy vegetables [23]. In this study, transcriptomic analysis of the green-leafed and purple-leafed varieties of Pak Choi was conducted using RNA-Seq technology. The current results not only identified Pak Choi’s vital structural genes involved in anthocyanin biosynthesis, but also revealed the potential regulatory mechanism involved in anthocyanin biosynthesis, transport and accumulation in the purple leafed variety.

One objective of this study is to identify the structural genes involved in anthocyanin biosynthetic pathway. The current result confirmed the presence of all known structural genes in this pathway in Pak Choi, which result was in agreement with previous proposal that anthocyanin biosynthesis pathway and structural genes are relatively conserved across higher plants [24]. Yet, at the transcriptional level, great variations were found among these structural genes between ‘P’ and ‘G’. In the variety ‘P’, most genes in flavonoid pathway had significantly higher expression levels. For examples, our results showed that the expression of *Bra017210* and *Bra039777*, encoding for *PAL1* and *PAL2* respectively, were both higher in ‘P’ than in ‘G’, suggesting that the phenylpropanoid pathway which is upstream of anthocyanin biosynthesis was more active in the purple leafed variety [25, 26]. *4CL2* was another enzyme catalyzing the formation of hydroxycinnamic acid derivatives [27], and our study also revealed that the homologous gene of *4CL2* was up-regulated in ‘P’.

In the anthocyanin biosynthetic pathway, structural genes can be classified into two types: Early Biosynthetic Genes (EBGs) and Late Biosynthetic Genes (LBGs) [28]. The EBGs, including *CHS*, *CHI*, *F3H*, *F3’H*, and *F3’5’H*, catalyze the production of flavonols and other flavonoid compounds, while the LBGs, including *DFR*, *ANS*/*LDOX*, and the UDP-glucose: flavonoid-3-O-glucosyltransferase (*UFGTs*), are specifically for anthocyanin biosynthesis [5, 29]. Our results showed that both early biosynthetic genes (*Bra037747* and *Bra029212*) and late biosynthetic genes (*Bra027457*, *Bra013652*, *Bra019350*, *Bra003021*, *Bra035004*, and *Bra038445*) concurrent with the high anthocyanin content in ‘P’. O-methyltransferase (OMT) plays important roles in catalyzing the methylation of anthocyanins [30]. And in ‘P’, the expression level of *Caffeic acid 3-O-methyltransferase 1* and *Caffeoyl-CoA O-methyltransferase 1* were both higher than those in ‘G’, while *Quercetin 3-O-methyltransferase 1* and *Caffeic acid 3-O-methyltransferase OS* were lower in ‘P’ than in ‘G’. Together, these results confirmed that most structural genes were up-regulated for biosynthesis of anthocyanin which was in accordance with previous studies [31]. On the other side, exceptions did exist for the expression of the anthocyanin structural genes. For example, *Bra029212* was expressed at a lower level in ‘P’ than those in ‘G’. We reasoned that the possible reasons for such exceptions were that these genes either did not directly participate in the flavonoid biosynthesis pathway, or their expressions were suppressed due to the high-expression level and compensation effect of their redundant allelic genes. Nevertheless, these structural genes in anthocyanin biosynthesis were associated with the level of anthocyanin contents in ‘P’ and ‘G’.

On the other hand, transcriptional regulation of the structural genes in anthocyanin biosynthesis pathway was an important regulatory strategy for anthocyanin formation and accumulation in Arabidopsis [28, 32]. These regulatory genes can be mainly classified into two groups: positive and negative regulatory genes. Unlike bHLH and WD proteins that may have broader and overlapping regulatory targets, MYB proteins are the key components providing specificity for the subsets of regulated genes [33]. It has been reported that three transcription factors (MYB11, MYB12, and MYB111) activate early biosynthetic genes of the anthocyanin biosynthetic pathway in Arabidopsis [34]. While in Pak Choi, there’s only one ortholog of *AtMYB111*, but no ortholog of *AtMYB11* and *AtMYB12* was found either due to the absence of these genes in the Pak Choi genome or because of their extremely low expression level in both of these two Pak Choi varieties. The *bHLH* gene family regulates anthocyanin biosynthesis through formation of MBW ternary complexes [35]. In Pak Choi, only one *bHLH* transcriptional factor (*TRANSPARENT TESTA8*, *TT8*) was found that potentially involved in the regulation of anthocyanin biosynthetic genes. In Arabidopsis, *TT8* not only regulates anthocyanidin production towards the synthesis of proanthocyanidins in seeds, but is also involved in the regulation of anthocyanin biosynthesis in vegetative tissues and cell cultures [35]. Our transcriptional result was also consistent with the functional role of *TT8* in Pak Choi. There are several transcription factors including two R3-type single MYB proteins (MYBL2 and CPC) and three members of the LBD (LATERAL ORGAN BOUNDARIES DOMAIN) family that act as negative regulators of activities of WBM complexes decreasing anthocyanin biosynthesis in *A. thaliana* [5, 36]. Yet, the corresponding orthologs in Pak Choi (*Bra016164* and *Bra039283*) showed higher expression levels in ‘P’ than in ‘G’, probably either due to the different but contradictory function of these two genes in Pak Choi or due to the feedback effect of the high anthocyanin content in ‘P’ that in turn inhibited their expressions.

The flavonoid transporters also involved in the vacuolar transport of anthocyanins and proanthocyanidin precursors and might contribute to the accumulation of anthocyanin in plant as well [37–39]. In Arabidopsis, three genes, *TT12*, *TT19* and *AHA10*, have been found to be related to the transport of anthocyanins. However, our results showed that *B*ra009033 was down regulated while genes encoding *ATPase 10* and *ATPase 1* were up-regulated in purple-leafed variety. Glutathione S-transferases (GSTs) act as non-enzymatic carrier proteins enabling intracellular shuttling of endogenous compounds such as anthocyanin in plants. Previous gene knockout experiments revealed that GSTs were involved in anthocyanin transport [40] and the deposition of anthocyanin pigment into the vacuole [41]. The differential expressions of the GST encoding genes were found in this study, indicating a possible link between GST and the transportation of anthocyanin in Pak Choi as well.

## Conclusions

We performed RNA-Seq for two varieties of Pak Choi with contrasting different anthocyanin contents. Nine structural genes in the anthocyanin biosynthetic pathway were up-regulated in the purple-leafed variety. Among them, the late biosynthetic genes including *Bra027457*, *Bra013652*, *Bra019350*, *Bra003021*, *Bra035004*, and *Bra038445,* probably played more pivotal roles in the biosynthetic process of anthocyanin. In addition, key transcription factor and transporter genes, such as *Bra016164* and *Bra009033*, were also identified for the regulation of anthocyanin accumulation and transportation. The results in this study paved groundwork for further functional study on anthocyanin-related genes that will ultimately decipher the mechanism underlying persistently high anthocyanin contents in certain varieties of Pak Choi, and such knowledge shall also be useful for the other leafy horticultural crops.

## Additional files

Additional file 1: Table S1. The primers used for qRT -PCR. (DOCX 15 kb)

Additional file 2: Differentially expressed genes (DEGs) between the two Pak Choi varieties. (XLSX 256 kb)

## Abbreviations

4CL: 4-coumaroyl:CoA-ligase
ANS/LDOX: Anthocyanidin synthase
bHLH: Basic Helix-Loop-Helix
C4H: Cinnamate 4-hydroxylase
CHI: Chalcone isomerase
CHS: Chalcone synthase
DEGs: Differentially expressed genes
DFR: Dihydroflavonol 4-reductase
DHK: Dihydrokaempferol
EBGs: Early Biosynthetic Genes
F3′5′H: Flavonoid 3′5′-hydroxylase
F3′H: Flavonoid 3′-hydroxylase
F3H: Flavanone 3-hydroxylase
FPKM: The expected number of Fragments *per* Kilobase of transcript sequence *per* Millions base pairs sequenced
GO: Gene Ontology
GSTs: Glutathione S-transferases
KEGG: Kyoto Encyclopedia of Genes and Genomes
LBD: Lateral Organ Boundaries Domain
LBGs: Late Biosynthetic Genes
OMT: O-methyltransferase
PAL: Phenylalanine ammonia-lyase
RT-qPCR: Real-time quantitative PCR
TT8: Transparent TESTA8
UFGTs: The UDP-glucose: flavonoid-3-O-glucosyltransferase
