# Supplementary material for: Transcriptomic profiling of two Pak Choi varieties with contrasting anthocyanin contents provides an insight into structural and regulatory genes in anthocyanin biosynthetic pathway
Source: BMC Genomics. 2017 Apr 11;18:288. doi: 10.1186/s12864-017-3677-7 (PMC5387373; doi:10.1186/s12864-017-3677-7)
Supplement: Supplementary file 1 — The primers used for qRT -PCR. (DOCX 15 kb) [file 12864_2017_3677_MOESM1_ESM.docx]

**Table S1** The primers used for qRT-PCR.

| **Gene** | **Forward primer (5’-3’)** | **Reverse primer (5’-3’)** |
| --- | --- | --- |
| Actin | AGCGTGAGGAAGAGCATAAC | GTTGCTATCCAGGCTGTTCT |
| Bra013652 | GCTTGCAGCTGTTCTACGAG | ACCAACCCACGGTGAAGTAT |
| Bra019350 | GAAGAGTTCTTCGGTTTGCC | ACCCTTGGATCTTTCCTGTG |
| Bra027457 | TCTTCTTGATTTGCCAAACG | CATCGTAGCTTCCTTCGTCA |
| Bra037747 | TTCTCTTGAAGCCGCCTTAT | GTTTATCCCTGACCTTGCGT |
| Bra017520 | TGACTTCGATGACGGTTAGC | TTTGCAACCCTAAATCACCA |
| Bra017523 | TTGAGACGCGAGAAGCTAAA | GACGCTTTGTCCATCTTTGA |
| Bra026967 | TTTGAGAAAGAACCGGGAGT | GGCCTTCAAGTTAGCCAAAG |
| Bra016164 | GTCTCGTCCCGATAAAGACC | GAGGATCCGCTAGGTTCTTG |
| Bra032635 | AAGTCGAAGCATCCCAAGAC | TCCCACTCAAGACTGCTCAC |
